# Molecular characterization of a family 5 glycoside hydrolase suggests an induced-fit enzymatic mechanism

**DOI:** 10.1038/srep23473

**Published:** 2016-04-01

**Authors:** Marcelo V. Liberato, Rodrigo L. Silveira, Érica T. Prates, Evandro A. de Araujo, Vanessa O. A. Pellegrini, Cesar M. Camilo, Marco A. Kadowaki, Mario de O. Neto, Alexander Popov, Munir S. Skaf, Igor Polikarpov

**Affiliations:** 1 São Carlos Institute of Physics, University of São Paulo, São Carlos 13566-590 São Paulo, Brazil; 2 Institute of Chemistry, University of Campinas, Campinas 13084-862, São Paulo, Brazil; 3 Institute of Bioscience, State University of São Paulo, Botucatu 18618-970, São Paulo, Brazil; 4 European Synchrotron Radiation Facility, Grenoble CS40220, France

## Abstract

Glycoside hydrolases (GHs) play fundamental roles in the decomposition of lignocellulosic biomaterials. Here, we report the full-length structure of a cellulase from *Bacillus licheniformis* (*Bl*Cel5B), a member of the GH5 subfamily 4 that is entirely dependent on its two ancillary modules (Ig-like module and CBM46) for catalytic activity. Using X-ray crystallography, small-angle X-ray scattering and molecular dynamics simulations, we propose that the C-terminal CBM46 caps the distal N-terminal catalytic domain (CD) to establish a fully functional active site via a combination of large-scale multidomain conformational selection and induced-fit mechanisms. The Ig-like module is pivoting the packing and unpacking motions of CBM46 relative to CD in the assembly of the binding subsite. This is the first example of a multidomain GH relying on large amplitude motions of the CBM46 for assembly of the catalytically competent form of the enzyme.

The production of biofuels from renewable sources is an important element of the global strategy for generating sustainable energy with reduced environmental impact. Current technologies for obtaining liquid biofuels and green chemicals rely on the enzymatic digestion of lignocellulosic biomass from a variety of feedstocks^1^. Plant biomass-the most abundant source of carbohydrates on Earth-is primarily composed of cellulose microfibrils surrounded by a hydrated heteropolymeric matrix of hemicellulose and lignin. Plant biomass may be subjected to thermo-chemical pretreatments and enzymatic reactions to produce soluble fermentable sugars.

The canonical model of hydrolytic degradation of cellulose requires at least three classes of enzymes^1^. Cellobiohydrolases (CBHs) processively cleave the glycosidic bonds at the reducing and non-reducing ends of cellulose chains in crystalline regions to produce cellobiose. Endoglucanases (EGs) introduce random cuts in the amorphous regions of cellulose and create new chain extremities for CBH attack; thus, these enzymes act synergistically. The released cellobiose molecules are then enzymatically converted into glucose by β-glucosidases.

The molecular architecture of glycoside hydrolases (GHs) frequently consists of a catalytic domain (CD), where hydrolysis occurs, and one or more ancillary modules (AMs), which are usually connected by less structured linkers. The most common type of AMs are carbohydrate-binding modules (CBMs), which are able to recognize and bind specific carbohydrate chains^2^. Generally distinct and independent structural domains, the CBMs facilitate carbohydrate hydrolysis by increasing the local concentration of enzymes at the surface of insoluble substrates, thereby targeting the CD component to its cognate ligands. CBMs might also disrupt the crystalline structure of cellulose microfibrils, although the underlying mechanism remains poorly understood. Thus, CBMs enhance the accessibility of CDs to carbohydrate chains to improve enzymatic activity, making them important candidates for the development of effective biomass-degrading enzymes in industrial settings.

Although there are examples of active GHs that lack AMs^3^,^4^, the majority of the enzymes depend on AMs for activity^2^. In several cases, CBMs were shown to extend and complement the CD substrate-binding site in multimodular carbohydrate-active enzymes, such as endo/exocellulase E4 from *Thermobifida fusca*^5^, chitinase B from *Serratia marcescens*^6^, a starch phosphatase from *Arabidopsis thaliana*^7^ and a GH5 subfamily 4 (GH5_4) endoglucanase from *Bacillus halodurans* (*Bh*Cel5B)^8^. A pioneer work of Sakon *et al.*^5^ revealed that rigid structural extension of the GH9 CD by a type C CBM3 imprints a processive mode of action to this endoglucanase. Further publications showed that CBM-based structural extensions of the active site are important for substrate engagement and recognition^7^,^8^,^9^.

Recently, Venditto *et al.*^8^ reported the X-ray structure of the tri-modular GH5_4 endoglucanase from *Bacillus halodurans* (31% sequence identity to BlCel5B), with the CBM46 extension of the active site appended to the CD via an immunoglobulin (Ig)-like module. Removal of the CBM46 caused a ~60-fold reduction of the activity of the enzyme against β-glucans, but showed little or no effect against xyloglucan hydrolysis. Moreover, the CBM46 mediated a significant increase in the *Bh*Cel5B activity in plant cell wall settings. Modeling of cellotriose in the negative subsites of the active site of *Bh*Cel5B demonstrated the structural conservation of the -1 position, but provided little information about direct interactions between CBM46 and the substrate. It was speculated that β-1,3 kink of the β-glucan might allow the ligand to reach for the CBM46, whereas pure β-1,4 linkages in the backbone of xyloglucan chains would restrict binding to the CD, thus explaining the lack of influence of the CBM46 on the enzymatic activity of *Bh*Cel5B against xyloglucans in solution^8^. It was also argued that the CBM46 could potentialize the activity by driving *Bh*Cel5B towards xyloglucan-rich regions in the context of the plant cell walls, but no large-scale conformational adjustments of the AMs have been shown to occur or suggested to take part in the enzymatic activity^8^.

The mechanisms of ligand binding mediated by large-scale conformational changes in proteins following the induced-fit^10^ or conformational selection^11^ models have recently attracted considerable attention^12^,^13^,^14^. Although initially introduced as contradictory theories, these two limiting cases can be unified considering the flux description concept^13^ or the extended conformational selection model^14^. While local ligand-induced conformational adjustments have been reported for carbohydrate-active enzymes^5^,^15^,^16^,^17^, cognate ligands recognition and hydrolysis mediated by a large-scale conformational mobility of distinct domains in multidomain settings is uncommon for endoglucanases.

Here, we report the crystal structure of a full-length GH5_4 enzyme from *Bacillus licheniformis* (*Bl*Cel5B) that exhibits two AMs (Ig-like module and CBM46) appended to the CD. We structurally and functionally characterize the enzyme using a combination of protein crystallography, small-angle X-ray scattering (SAXS), molecular dynamics computer simulations and site-directed mutagenesis, and show that the AMs and their conformational mobility are essential for the enzymatic activity of *Bl*Cel5B. We find that the large-scale conformational adjustments of the distal CBM46 mediated by the Ig-like hinge domain are crucial in active-site assembly for optimal substrate binding and hydrolysis. We propose that the *Bl*Cel5B conformational selection/induced-fit mechanism of hydrolysis represents a novel paradigm that applies to several GH5_4 members and, possibly, to a number of other multidomain GHs.

## Results

### *Bl*Cel5B Crystal Structure

*Bl*Cel5B crystals in the substrate-free form and complexed with cellopentaose (C5) were obtained and diffracted to 1.7 Å and 1.75 Å resolutions, respectively (Supplementary Table 1). The substrate-free and complexed structures exhibited no substantial conformational differences (with the exception of the substrate). Because of minor variations in the loops located distal to the substrate-binding site, a root mean squared deviation (rmsd) of 0.33 Å between the complexed and substrate-free structures was observed. A single protein chain occupies the asymmetric unit, and most of the residues were built, with the exception of the first 17 residues and those in the loop between L398 and P405 due to weak electron density.

**Figure 1:**
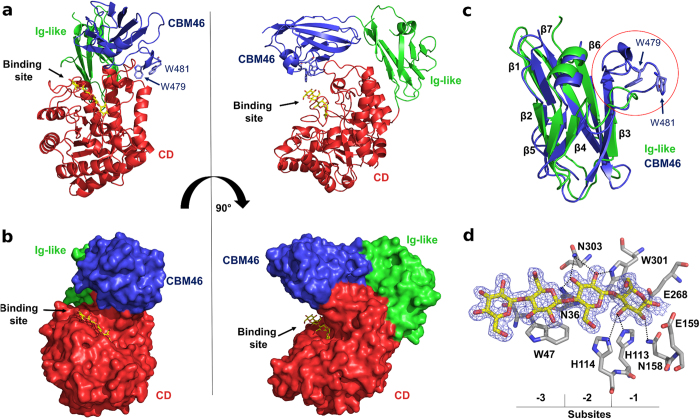
Crystal models of *Bl*Cel5B. Complete structure is shown as a cartoon illustration in (**a**) and a van der Waals surface in (**b**). The CD module (red) has a typical TIM-barrel fold, and its substrate-binding site is adjacent to CBM46 (blue). Despite the proximity of the binding site in the crystallographic model, the CBM46 residues W479 and W481 are distant from the substrate cellotetraose (yellow). The Ig-like domain (green) has a lateral position, serving as a connector between the CD and CBM46. (**c**) A superposition of the Ig-like domain and CBM46 illustrates their structural similarity, with most of the structural differences present in the loop highlighted by a red circle. (**d**) Cellotetraose occupies subsites -1 to -3 and is primarily coordinated by the residues represented in gray.

The *Bl*Cel5B structure comprises three distinct domains: an N-terminal CD (residues 18 to 330), an Ig-like module (residues 335 to 428) and a family 46 CBM (residues 432 to 533) (Fig. 1A,B). Similarly to other members of the GH5 family, the CD of *Bl*Cel5B has a typical TIM barrel fold with eight inner β-strands and eight outer α helices that are interconnected by loops and three short α helices. Very short linkers, D429-D430-P431 and V331-P332-N333-A334, connect the CBM46 to the Ig-like module and the Ig-like module to the CD, respectively. Both Ig-like module and CBM46 have a β-sandwich fold composed of two β-sheets of four and three antiparallel β-strands interconnected by loops and a short α helix between strands β3 and β4 (Fig. 1C). A structural comparison between the Ig-like module and the CBM46 using the Dali server^18^ yielded an rmsd of 2.3 Å and a Z-score of 10.2. However, despite their structural resemblance, these modules share only 17% sequence identity. A structure-based search performed using the same server showed that the Ig-like module is similar to the Ig-like module from a recently solved crystal structure of a tri-modular GH5_4 enzyme from *Bacillus halodurans*^8^*, Bh*Cel5B, with rmsd = 1.3 Å and Z-score = 15.3. The CBM46 from *Bh*Cel5B is the most structurally similar to *Bl*Cel5B CBM46, with rmsd = 1.6 Å and Z-score = 12.4. The sequence identity relative to *Bh*Cel5B, however, is low (28% for Ig-like and 25% for CBM46).

The Ig-like module, adjacent to the CD, contains only one tyrosine (Y367) exposed to solvent and no tryptophan residues. Because aromatic residues play a major role in glucose recognition, this observation suggests that substrate binding may not be the primary function of Ig-like module. In contrast, the CBM46 has three tryptophan residues, two of which face the CD substrate binding site (Fig. 1A), indicating that it may be actively engaged in the carbohydrate binding.

**Figure 2:**
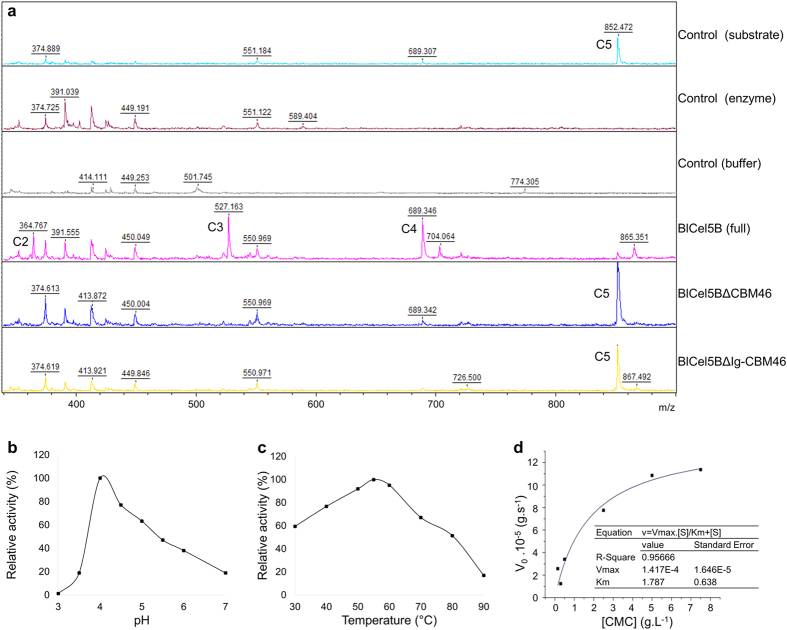
*Bl*Cel5B enzymatic activity characterization. (**a**) MALDI/TOF-MS spectra of the products released after incubation of *Bl*Cel5B and its two deletion constructs (ΔCBM46 and ΔIg-CBM46) with the substrate cellopentaose (C5). The first three spectra show the substrate, enzyme and buffer controls. The forth spectrum reveals that full length *Bl*Cel5B is capable of enzymatic hydrolysis of C5 into smaller oligosaccharides such as C4, C3 and C2. The last two spectra show that the C-terminal deletions eliminate the enzyme activity. *Bl*Cel5B activities on CMC as functions of pH and temperature are shown in (**b**) and (**c**), respectively. The enzyme exhibits optimal pH of 4.0 and optimal temperature of 55 °C, retaining about 50% of its activity at 80 °C. (**d**) Michaelis-Menten curve using CMC as a substrate.

Electron density maps clearly reveal the presence of a cellotetraose (C4) and not a soaked cellopentaose (C5) in the CD negative substrate-binding subsites (Fig. 1D), indicating that *Bl*Cel5B is catalytically active in the crystal state and able to cleave a C5 molecule. The lack of electron density verifies the absence of the fifth glucose moiety from the soaked C5, and a closer inspection of the structure confirmed that the presence of a fifth glucose unit would be sterically hindered by the catalytic residues on the reducing end and by residue R234 of a symmetry-related enzyme molecule on the non-reducing end. The ability of *Bl*Cel5B to cleave C5 into glucose and C4 molecules in solution was demonstrated by enzymatic product profile mass spectrometry analysis (Fig. 2A). The C4 oligomer in the *Bl*Cel5B binding site is coordinated by hydrogen bonds to residues N36, H113, H114, N158, W301, and N303 and by a CH-π interaction with residue W47 (Fig. 1D). These residues belong to the CD and are conserved in the GH5 family.

### *Bl*Cel5B enzymatic activity

*Bl*Cel5B exhibits optimum activity toward carboxymethylcellulose (CMC; 8.7 U/mg) at a pH of 4.0 and 55 °C and retains approximately half of its maximum activity at 80 °C, demonstrating considerable thermal stability (Fig. 2B,C). *Bl*Cel5B is also active on β-glucan (34 U/mg), lichenan (17.8 U/mg) and xyloglucan (15.7 U/mg) substrates (Table 1), whereas no activity was detected on galactomannan, rye arabinoxylan, 1,4-β-mannan or the insoluble substrate Azo-Avicel. Kinetic parameters were calculated assuming Michaelis-Menten behavior with CMC as substrate: *K*_*M*_ = 1.78 g L^−1^ and *V*_*max*_ = 1.41 × 10^−4^ g s^−1^ mg protein^−1^ (Fig. 2D). Although *Bl*Cel5B is not a highly active enzyme against one specific substrate as compared to others GH5_4^19^, it has the advantage of being active against different substrates with β-1,3 and/or β-1,4 glycosidic linkages.

To understand the importance of the ancillary modules for *Bl*Cel5B activity, enzymatic assays were carried out using four enzyme mutants: a CBM46 deletion (ΔCBM46) and an Ig-like + CBM46 deletion (ΔIg-CBM46) as well as point mutations of the CBM46 inner surface residues W479A and W481A. These mutants were expressed and purified as described for the wild-type enzyme. Strikingly, neither of the deletion variants exhibited detectable activity toward any of the substrates tested using full-length *Bl*Cel5B (Table 1), demonstrating that the Ig-like module and the CBM46 are essential for *Bl*Cel5B activity. Thermal shift assays were conducted to confirm structural stability of the mutants (Supplementary Fig. 1). All of the constructs showed similar melting temperatures: 62 °C for *Bl*Cel5B, 58 °C for *Bl*Cel5BΔCBM46, 56 °C for *Bl*Cel5BΔIg-CBM46, 65 °C for *Bl*Cel5BW479A and 59 °C for *Bl*Cel5BW479A, thus confirming their proper overall fold.

We also examined the function of the CBM46 inner surface residues W479 and W481 (Fig. 1A) in *Bl*Cel5B activity by performing enzymatic assays with W479A and W481A mutants. Both mutations reduced enzymatic activity toward all tested substrates (Table 1), with W481A having a stronger effect than W479A (~64% vs. 79% activity relative to wt *Bl*Cel5B using β-glucan and ~10% vs. 50% using CMC). This indicates that CBM46 must interact with the substrate via residues W479 and W481. However, since the *Bl*Cel5B crystal structure exhibits no close contact between these residues and the substrate, these results suggest the existence of large-amplitude interdomain motions that may enable direct interactions between CBM46 and the carbohydrate.

### *Bl*CelB5 dynamics and binding-site architecture

Molecular dynamics (MD) simulations were performed to investigate the conformational mobility of *Bl*Cel5B. In the simulations of the crystal structure for *Bl*Cel5B bound to C4, the substrate dissociates from the protein within the first 100 ns of the simulation time (Supplementary Fig. 2A). This observation suggests that cellotetraose does not exhibit detectable affinity for this specific *Bl*Cel5B conformation in solution, as one might otherwise expect for a reaction product. No changes beyond local fluctuations were observed in any of the three *Bl*Cel5B domains within the time scale of these runs (400 ns; Supplementary Fig. 2B). However, the CBM46 and Ig-like domains did exhibit rigid body-like motions relative to the CD, with rmsd values around 2.3 Å and 1.8 Å, respectively, suggesting that *Bl*Cel5B may execute large-amplitude interdomain motions over longer time scales (Supplementary Fig. 2B,C).

**Figure 3:**
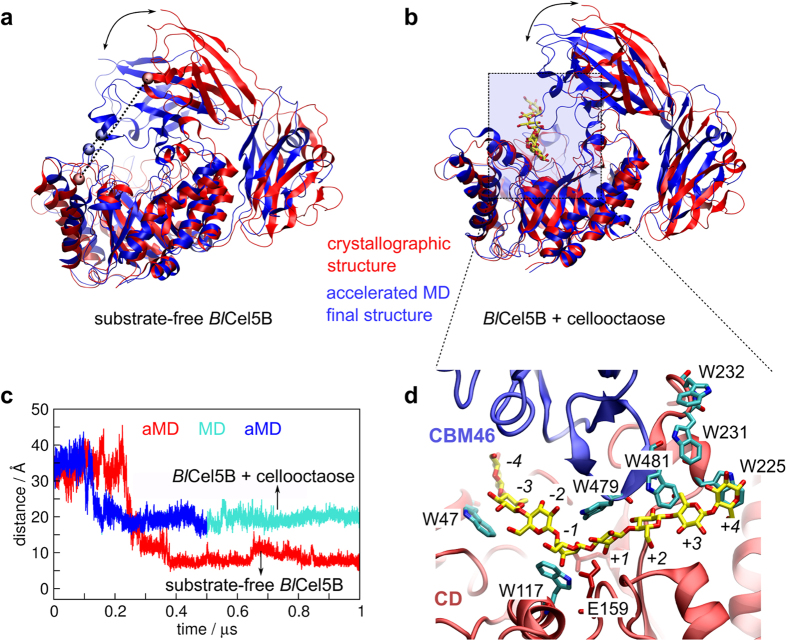
Open-close transitions of *Bl*Cel5B. (**a**) *Bl*Cel5B in the absence of substrate and (**b**) in the presence of cellooctaose, as observed in our aMD simulations. The distance between the α carbon of residues I120 (CD) and E477 (CBM46), illustrated as spheres in (**a**), is plotted in **(c)**, revealing a transition by the decrease in the distance from 40 Å to 7 Å (substrate-free) or 20 Å (in presence of cellooctaose). For the substrate-free enzyme, the red line refers to a 1 μs-long aMD; for the *Bl*Cel5B-cellooctaose complex, the first 500 ns refers to aMD (in blue) and the second 500 ns to conventional MD (in turquoise). (**d**) A snapshot of the *Bl*Cel5B-cellooctaose complex, highlighting the tryptophan residues that interact with the glucan chain in subsites −4 to +4. Residues W479 and W481 belong to CBM46 and only become available for substrate interactions in the closed configuration of *Bl*Cel5B.

Accordingly, simulations were then performed using accelerated molecular dynamics (aMD) techniques to probe *Bl*Cel5B interdomain motions. aMD enhances conformational sampling by raising the basins of the dihedral potential energy surface without affecting the general form of the atomistic potential, thereby increasing transition rates between different local minima. aMD trajectories corresponding to more than 1.0 μs of conventional MD runs were generated^20^. During these simulations, we observed occlusive conformations between CBM46 and CD that resulted in a rearrangement of the enzyme’s architecture around the active site (Video S1). Figure 3A shows *Bl*Cel5B in the crystallographic conformation (red) and in a selected configuration obtained with aMD (blue) in the absence of the substrate. Interdomain motions were gauged by the time evolution of the distance between the α carbons of residues I120 and E477 (represented as spheres in Fig. 3A), belonging to the CD and CBM46, respectively. Figure 3C shows that the I120-E477 distance (red curve) gradually decreases from ~35 Å to ~7 Å within the first half of the 1.0 μs aMD trajectory, indicating a transition between the semi-open (crystallographic) and occluded (aMD sampled) configurations. During the second half of the aMD simulation, the full-length enzyme remained in the closed conformation, with the CBM46 covering the carbohydrate-binding site. These results suggest that *Bl*Cel5B undergoes large-scale interdomain movements that enable interactions between CBM46 and the substrate bound to the CD.

To study the interactions of *Bl*Cel5B with a non-hydrolyzed glucan chain, we built a model structure with a cellooctaose (C8) chain spanning the entire positive (+1 to +4) and negative (−4 to −1) subsites of the enzyme. Starting from the crystallographic *Bl*Cel5B conformation, the C8 molecule deviated significantly from the active site and assumed a non-productive binding mode (Supplementary Fig. 2D). This observation suggests that the open conformation of *Bl*Cel5B is not able to hold the substrate in a position suitable for hydrolysis (Supplementary Fig. 2E). However, after subjecting the *Bl*Cel5B-C8 complex to a 0.5 μs aMD simulation with harmonic restraints on the C8 chain to prevent it from deviating from the productive binding mode, the CBM46 readily closed over the CD and trapped the C8 chain in position for hydrolysis (Fig. 3B). In the presence of the substrate, CBM46 adopts a final conformation intermediate between the crystallographic structure and that observed in the substrate-free *Bl*Cel5B aMD simulations; this is illustrated by the I120-E477 distance, which stabilizes near 20 Å in the closed configuration that traps the C8 molecule (in contrast to ~7 Å for substrate-free *Bl*Cel5B) (Fig. 3C). This *Bl*Cel5B-C8 configuration remains stable over an additional 500 ns of conventional MD simulation with no restraints (Fig. 3C cyan line, Supplementary Fig. 2E,F).

A closer inspection of the productive binding mode obtained from these extensive simulations reveals that the CBM46 tryptophan residues W479 and W481 (along with CD tryptophan residues) play important roles in carbohydrate recognition and orientation by creating a tunnel-like topology along the *Bl*Cel5B binding cleft, as depicted in Fig. 3D. Together, these results indicate that CBM46 is a key component of the catalytic active complex, providing an explanation as to why CBM46 is essential for the enzymatic activity of *Bl*Cel5B.

**Figure 4:**
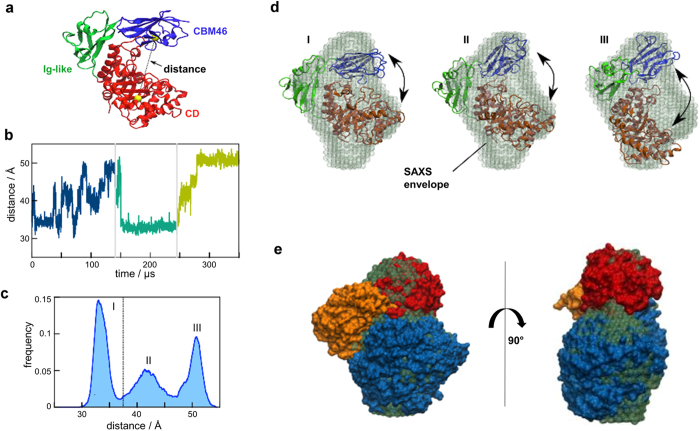
Large-scale movements of *Bl*Cel5B modules and superposition of their representative conformations with the SAXS envelope. (**a**) *Bl*Cel5B structure showing the distance between the backbone beads of residues I120 and E477, which are centrally located in CD and CBM46, respectively, as a metric for the relative disposition between the two domains. (**b**) Time history of the I120-E477 distance computed using CG-MD simulations. Different colors separated by vertical lines correspond to independent simulations of approximately 120 μs. (**c**) The distance distribution indicates three major peaks: closed or occluded CBM46/CD conformations (I); semi-open (II), which is similar to the crystallographic structure; and extended conformers (III). (**d**) Superimposition of the three representative molecular conformations of *Bl*Cel5B with the SAXS model. (**e**) Average structures obtained from the simulation segments corresponding to population groups I-III, which are individually superposed on the SAXS envelope.

To enable substantially longer time scales compared to atomistic simulations, we further explored the dynamics of *Bl*Cel5B using coarse-grained MD (CG-MD) simulations. We performed three independent ~120 μs CG-MD simulations, for a total of approximately 360 μs of sampling. The distance between the α carbons of two residues centrally positioned in the CD and CBM46 (Fig. 4A) was monitored, and the results shown in Fig. 4B indicate that the wide-amplitude events described above frequently appear in this time scale. The computed distance distribution depicted in Fig. 4C indicates three main conformational states ranging from (I) closed conformations similar to those encountered in the substrate-free aMD simulations, in which CBM46 interacts with the CD to shape the substrate binding site, to (II) semi-open conformations similar to the crystallographic structure, and (III) extended *Bl*Cel5B conformations in which the CD and CBM46 are even further apart than in the crystal structure.

### *Bl*Cel5B conformers fit the SAXS envelope

SAXS experiments were conducted to assess *Bl*Cel5B conformational states in solution, and the results revealed the enzyme in its monomeric form, with average values of *R*_g_ = 27.17 Å and *D*_max_ = 87.59 Å (Supplementary Table 2). The *ab initio* dummy atom model (DAM) demonstrated that the SAXS-derived *Bl*Cel5B molecular envelope could not be single-handedly filled by any of the main conformational states encountered in the simulations (Fig. 4D).

It is known that a Kratky plot exhibits a peak with an elevated baseline at high *q* for a monodisperse system composed of multi-domain particles with flexible extensions^21^,^22^. Indeed, an elevation of the baseline toward a hyperbolic-like curve was observed for *Bl*Cel5B, indicating a considerable degree of molecular mobility in solution (Supplementary Fig. 3). Thus, the conformational heterogeneity of the enzyme can be decomposed in structural terms as a combination of conformational states identified in our crystallographic and MD studies. We found that the SAXS envelope can be well represented by considering the superimposition of three different representative molecular conformations of *Bl*Cel5B (Fig. 4E): a closed or CBM46/CD-occluded conformation extracted from the simulations with a relative weight of 26%, a semi-open conformation represented by the crystal structure corresponding to 40%, and an extended conformation based on simulations that is responsible for 34% of the SAXS envelope. The resulting average scattering curve from this model fits the experimental protein scattering intensity, with χ = 1.89 (Supplementary Fig. 3).

### GH5_4 phylogenetic analysis

To date, there are 427 sequences classified as subfamily 4 members in the CAZy database^23^. After the exclusion of partial sequences and the suppression of highly identical members (higher than 90% identity), 144 sequences containing between 277 and 400 residues were aligned and used to construct a phylogenetic tree (Supplementary Fig. 4A). According to PFAM database^24^ conserved domain classification, 128 GH5 enzymes have an architecture consisting of an N-terminal catalytic module, a CBM_X2 module and an unknown module of approximately 100 residues at the C-terminus (Supplementary Fig. 4B). Of these, 12 enzymes have an additional CBM1, and 5 have a CBM2 at the N-terminal region. Based on this PFAM architecture and CAZy subfamily classification, all the 144 enzymes (including *Bl*Cel5B) belong to the GH5_4 subfamily and group together in the same branch of the phylogenetic tree, evidencing a common ancestor. These results support the hypothesis that the enzymes may employ the same mechanism by which ligand binding is mediated by an extensive conformational breathing of the enzyme that involves the large-scale movement of CBM46 around the Ig-like module (CBM_X2) as a structural hinge.

## Discussion

Growing interest in biotechnological applications of enzymes exhibiting activity toward lignocellulosic biomass has sparked efforts in the discovery and development of novel enzymes, as well as the search for a deeper understanding of their mechanisms of action. Here, we elucidate the trimodular molecular architecture of the full-length *Bl*Cel5B, a member of the GH5_4 subfamily, for which large-scale conformational dynamics appears to play a central role in its enzymatic activity. Full-length *Bl*Cel5B is active on both cellulosic and hemicellulosic substrates and auxiliary modules are crucial for its activity.

Most carbohydrate-active enzymes are modular and consist of a catalytic domain appended to one or more separate AMs. AMs, such as CBMs, typically recognize carbohydrates and target their cognate catalytic domains toward the substrate. Because the structural analysis of the protein is challenging if the linkers connecting the structural subunits of the enzyme are long and flexible, the standard approach is to study the domains separately. In this work, a combination of protein crystallography, computational molecular dynamics, and SAXS analyses enabled the identification of a new conformational selection-based molecular mechanism that involves GH5 catalytic domain and two AMs in full-length *Bl*Cel5B. We observed that the *Bl*Cel5B distal CBM46 is directly involved in shaping the local architecture of the substrate-binding site. Although the CD alone appears unable to bind the substrate for catalysis, the AMs exhibit open-close motions that allow the substrate to be captured in a suitable position for hydrolysis. Here, we advocate that large-amplitude motions of AMs are crucial for assembling the enzyme into its active conformation, highlighting a new function of CBMs. This mechanism of substrate binding closely resembles the extended conformational selection model^13^,^14^, with the induced-fit mechanism of reaction^10^ as its limiting case. To the best of our knowledge, this enzymatic mechanism has not been proposed previously for any GH.

**Figure 5:**
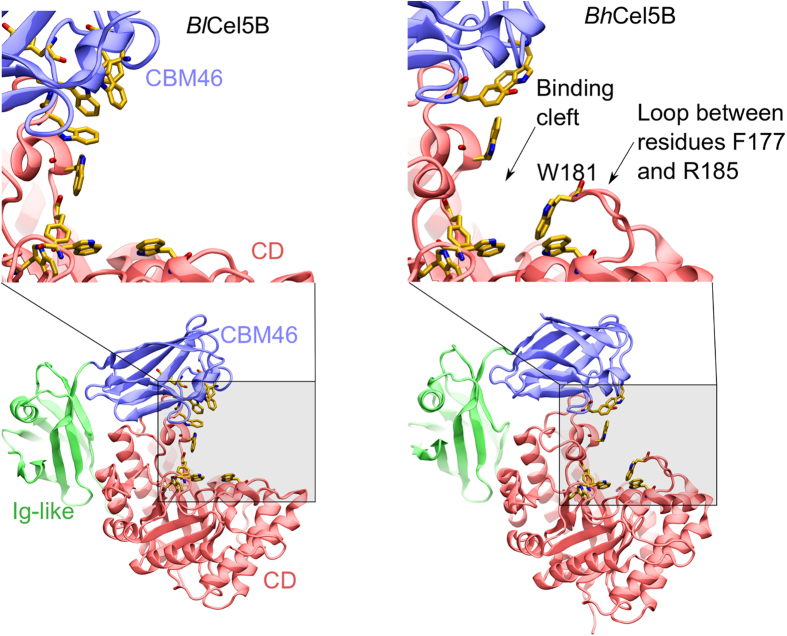
Comparison of the binding site shape of GH5_4 enzymes available on the Protein Data Bank. (**a**) *Bl*Cel5B in the crystallographic and closed configuration; (**b**) *Bacillus halodurans* Cel5B (*Bh*Cel5B) (PDB id: 4V2X) (**c**) *Piromyces rhizinflata* GH5 endoglucanase (PDB id: 3AYR); (**d**) *Clostridium cellulolyticum* GH5 endoglucanase (PDB id: 1EDG); (**e**) *Clostridium cellulovorans* GH5 endoglucanase (PDB id: 3NDY); (**f**) *Bacteroides ovatus* GH5 xyloglucanase (PDB id: 3ZMR); (**g**) *Paenibacillus pabuli* GH5 xyloglucanase (PDB id: 2JEP); (**h**) *Prevotella bryantii* GH5 endoglucanase (PDB id: 3VDH); (**i**) *Ruminiclostridium thermocellum* multifunctional GH5 cellulase, xylanase and mannase (PDB id: 4IM4); (**j**) *Bacteroidetes bacterium* AC2a endocellulase (PDB id: 4YHE).

**Figure 6:**
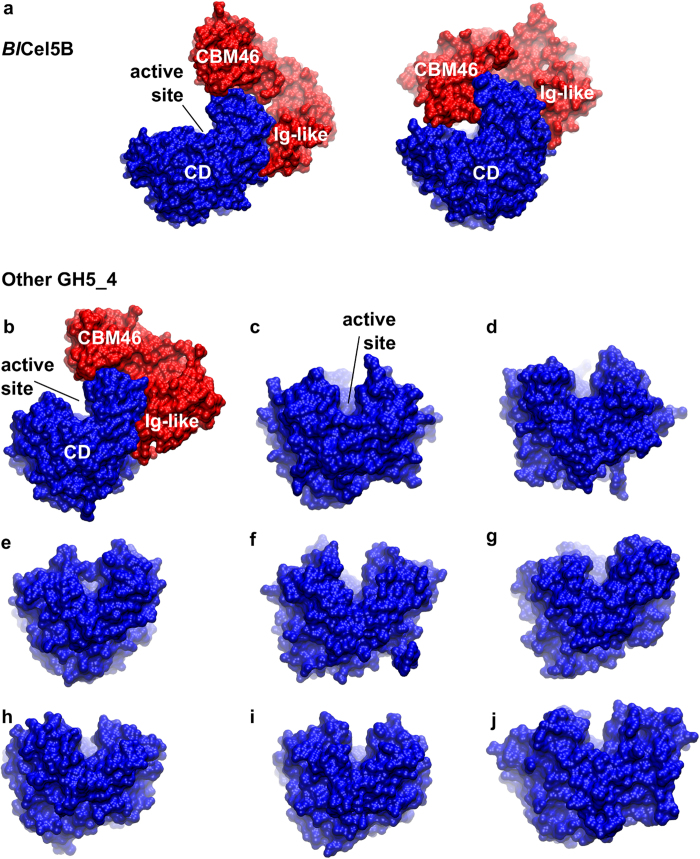
Comparison of the binding cleft of the *Bl*Cel5B and *Bh*Cel5B. The main difference between *Bl*Cel5B and *Bh*Cel5B is that the latter exhibits a deeper cleft due to the presence of residue W181 in the loop between F177 and R185. We conjecture that this difference in the binding site architecture relates to the importance that the CBM46 plays in the BlCel5B enzymatic mechanism.

The CD binding site of *Bl*Cel5B is open and relatively flat and is thus barely able to properly hold the substrate in position for catalysis without assistance from the CBM46. In contrast, other GH5s belonging to subfamily 4 listed in the Protein Data Bank^19^,^25^,^26^,^27^,^28^ exhibit a deep binding cleft or tunnel that can effectively entrap the substrate for catalysis (Fig. 5). Due to the marked interdomain conformational rearrangement observed in our simulations, the CBM46 generates a confined binding site in *Bl*Cel5B that resembles the binding site architecture of the other GH5 enzymes that lack AMs. Thus, *Bl*Cel5B appears to have adopted a strategy of CBM46-mediated interactions for proper functioning. Although the homologous *Bh*Cel5B has the same domain architecture of *Bl*Cel5B and belongs to the same subfamily (a comparison of the sequence and structure of *Bl*Cel5B and *Bh*Cel5B is presented in Supplementary Fig. 5), its binding site exhibits important differences that may impact the catalytic mechanism. The *Bh*Cel5B binding site is V-shaped and deeper than the *Bl*Cel5B binding site (Figs 5 and 6). This is due to the loop between residues F177 and R185 from *Bh*Cel5B (absent in the *Bl*Cel5B), which contains residue W181 that forms part of the binding cleft (Fig. 6). Consistently, although *Bh*Cel5B CBM46 is important for β-1,3-1,4-glucan hydrolysis (*Bh*Cel5B is about 60-fold less active without CBM46), the truncated enzyme is completely active against xyloglucan^8^, suggesting that the CBM46, in this case, is necessary for the binding to specific substrates. A closer inspection of results of the phylogenetic analysis, more specifically of the clade composed by GH5_4 enzymes with trimodular architecture (Supplementary Fig. 4C), reveals subclades whose main characteristic is the varying length of the loop located between residues 161 and 163 (*Bl*Cel5B residue numbering). Therefore, our results show that *Bl*Cel5B represents a smaller group of enzymes that are completely dependent on its AMs for hydrolysis of plant cell wall polysaccharides, and that the underlying mechanism may rely on large-scale interdomain motions.

The amino acid sequence of the *Bl*Cel5B Ig-like module is recognized by BLASTP as belonging to CBM_X2, a poorly described group that has been compared with CBM-like accessory modules without a defined function^29^. Despite the similarity of *Bl*Cel5B Ig-like module to CBMs, it lacks an identifiable aromatic residue-rich carbohydrate-binding site. Nonetheless, according to our results, the Ig-like module seems to play an important function as a structural hinge, dynamically holding the CBM46 and CD in positions that are appropriate for enzymatic activity.

**Figure 7:**
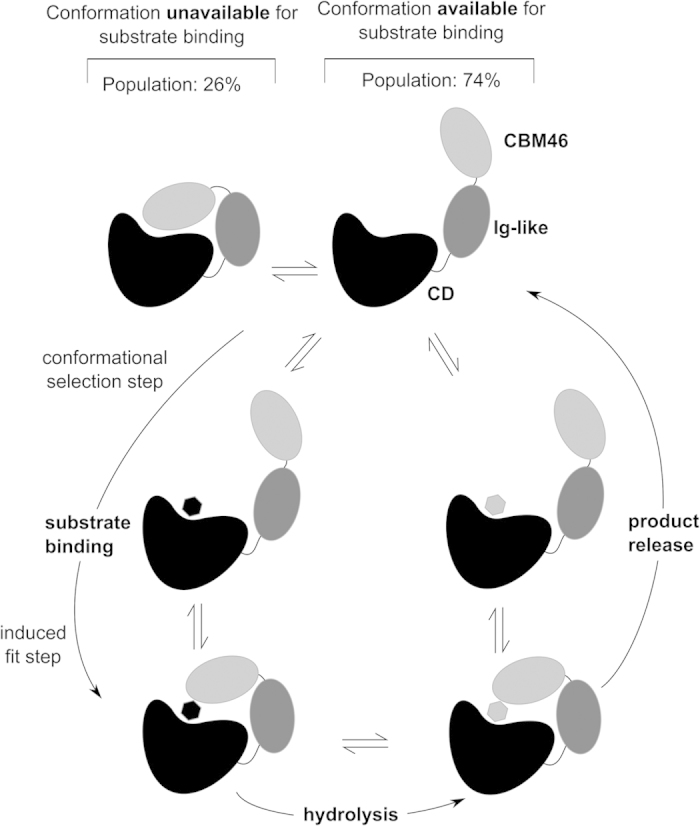
Proposed molecular mechanism of *Bl*Cel5B conformational selection. As suggested by the simulations and SAXS data, *Bl*Cel5B spans multiple conformations ranging from closed to extended CBM46/CD states. In a given open state, the substrate may reach the active site and become entrapped by the capping of CBM46 onto CD and induced-fit conformational adjustments. After hydrolysis, the reaction product is released to yield apo-*Bl*Cel5B, which becomes ready for a new cycle.

Based on the results of our crystallographic, computer simulation, and SAXS structural analyses, as well as site-directed mutagenesis and activity assays, we propose a molecular mechanism for *Bl*Cel5B substrate binding, which might apply to other GH5_4 subfamily enzymes that share this tri-modular architecture. *Bl*Cel5B can be found in several different conformational states ranging from CBM46/CD closed (or occluded) to extended conformations (Fig. 7). In extended configurations, the substrate may dock at the shallow substrate binding site of CD in one of the semi-closed conformations of the enzyme; however, its binding is properly stabilized for hydrolysis only with the aid of induced-fit repositioning mediated by CBM46. After cleavage, the intrinsic dynamics of *Bl*Cel5B would eventually allow the opening of the active site for product release. The proposed mechanism is consistent with our mutagenesis and enzymatic activity assays, which show that the Ig-like module and CBM46 are indispensable for *Bl*Cel5B catalytic activity and, together with the CD, form the unique catalytic domain of the enzyme. These experiments reveal a novel function for CBMs in which they are intimately involved in the assembly of the active site and catalytic process. Computer simulations suggest that large-scale motions of the CBM46 and Ig-like domains mediate conformational selection and final induced-fit adjustments to trap the substrate at the active site and promote hydrolysis. SAXS data support the modeling results, providing compelling evidence for highly mobile domains in solution.

## Methods

### Cloning, Expression and Purification

The gene encoding *Bl*Cel5B (GenBank: AAU23417.1) was amplified from *Bacillus licheniformis* genomic DNA (ATCC 14580) without the predicted signal peptide sequence (nucleotides 1 to 81) using the primers *Bl*cel5B_Fw and *Bl*cel5B_Rv (Supplementary Table 3). The fragment was cloned into the expression vector pETTRXA-1a/LIC by ligation-independent cloning (LIC), as described elsewhere^30^.

The same method was used for construction of domain deletions. For Ig-like + CBM46 deletion, Δ(Ig-CBM46), the fragment encoding the CD (nucleotides 82 to 1086) was amplified using the primers *Bl*cel5B_Fw and *Bl*cel5BΔ1087-1683_Rv. For CBM46 deletion, ΔCBM46, the fragment encoding the CD + Ig-like (nucleotides 82 to 1377) was amplified using the primers *Bl*cel5B_Fw and *Bl*cel5BΔ1378-1683_Rv (Supplementary Table 3). Both fragments were cloned into pETTRXA-1a/LIC.

The wt protein *Bl*Cel5B, mutated proteins and AM deletions were expressed in *E. coli* Rosetta2 (DE3) strain. The cells were grown at 37 °C and 150 RPM in Luria Bertani Broth medium supplemented with 50 μg/mL kanamycin to an A_600_ of 1.5–2.0, after which the temperature was reduced to 20 °C and protein expression was induced with 1 mM IPTG for 6 h.

The extract was then loaded onto a NiNTA resin (Qiagen) equilibrated with a washing buffer (5 mM imidazole, 100 mM NaCl, 50 mM Tris-HCl, pH 8.0). Non-absorbed material was washed with ten times column volume with washing buffer and the purified protein was eluted with 200 mM imidazole, 100 mM NaCl, 50 mM Tris-HCl at pH = 7.0. His6 tag was removed by overnight digestion with TEV (Tobacco Etch Virus) at 4 °C, and untagged protein was purified by gel filtration through a HiLoad 16/60 Superdex 200 column in buffer containing 50 mM NaCl, 25 mM Tris-HCl at pH 7.0.

### Site-directed Mutagenesis

The *Bl*Cel5B point mutations W479A and W481A were obtained by the inverse PCR method of site-directed mutagenesis^31^. Phusion^®^ “High-Fidelity” DNA polymerase (NEB, USA) was used for amplifications with the plasmid pETTRXA-1a/LIC-*Blcel5B* as a template. Mutagenic primers *Bl*cel5BW479A_Fw/Rv and *Bl*cel5BW481A_Fw/Rv (Supplementary Table 3) were generated by HTP-OligoDesigner tool (http://www.ifsc.usp.br/htpoligo/).

### Activity Assays

Enzymatic activity assays were performed by a colorimetric method using the 3,5-dinitrosalicylic acid (DNS)^32^, with glucose being a standard for the calibration curves. Assays of optimal temperature and pH were performed in triplicate with 1% medium-viscosity CMC as the substrate. For optimal temperature, the reaction mixture containing 10 μL of enzyme at 0.1 mg/mL, 50 μL of 1% (w/v) CMC and 40 μL of 50 mM sodium citrate buffer (pH 5.0) was incubated at 30 to 80 °C for 15 min and stopped by adding 100 μL of DNS solution. After this, the mixture was incubated again for 5 min at 100 °C and the absorbance was measured at 540 nm with a spectrophotometer. For optimal pH determination, the same amount of enzyme and substrate were diluted in 40 mM acetate/borate/phosphate buffer (ABF) with different pH values ranging from 2.0 to 10.0. The reactions were carried out under the predetermined optimal temperature.

The substrate specificity of the enzyme was determind using rye arabinan, xyloglucan, β-glucan, galactomannan, lichenan, β-mannan, Azo-Avicel and CMC as substrates. The substrates were diluted in water to 1% (w/v), and the reaction mixture was composed of 10 mL of purified enzyme at a concentration of 0.1 mg/mL, 0.4 mL of 50 mM sodium citrate buffer at pH 5.0, and 0.5 mL of 1% (w/v) substrate aqueous solution. The reaction was incubated at 50 °C for 15 min, followed by treatment with DNS as mentioned above. Enzyme unit was defined as the amount of enzyme that produces 1.0 μM of glucose in one minute for each substrate.

The kinetic parameters were determined by increasing concentrations of CMC. Reactions were performed in 50 mM sodium citrate buffer (pH = 4.0) at 50 °C, and measured by DNS method as well. Kinetic constants were determined by non-linear regression using OriginPro 8.0.

### Thermal Shift Assays

The thermal denaturation assays were performed using a Real Time PCR Machine (Stratagene Mx3005P) as described by Dupeux and co-workers^33^. Briefly, the enzymes were diluted to 10 μM in 50 mM sodium citrate buffer (pH = 4.0) containing 1x SYPRO orange dye (Thermo Fisher Scientific). The fluorescence emission of the probe was monitored (excitation and emission at 492 and 516 nm, respectively) varying the temperature between 25 and 75 °C with the rate of 1 °C/min.

### Cellopentaose Cleavage Experiment

The full-length *Bl*Cel5B and AM deletion constructs were tested for product formation from cellopentaose. Cellopentaose (1.0 mM) was incubated with 25 μg of purified enzyme in 10 mM ammonium bicarbonate buffer (pH 7.0) in a total volume of 50 μL. The reaction was incubated for 90 min at 50 °C and then stopped by treatment at 100 °C for 5 min. After centrifugation for 10 min at 16,000 g the samples were subjected to MALDI/TOF-MS. Samples were supplemented with NaCl to a final concentration of 20 mM and 1 μL of the supernatant was co-crystallized with 1 μL 2,5-dihydroxybenzoic acid (10 mg/mL) in acetonitrile 30% and spotted on the target plate. The products were analyzed on Microflex LT MALDI-TOF (Bruker Daltonics) operating in positive ion mode. A single spectrum was obtained by averaging four independent spectra generated by 300 laser shots at 60% potency.

### Crystallization, Data Collection, and Structure Determination

After purification, *Bl*Cel5B was concentrated to 10 mg/mL for crystallization trials. Crystallization screens were set up using the sitting-drop vapor-diffusion method on a Cartesian PixSys 4200 (Genomic Solutions, United Kingdom) in a 96-well plate with drops formed by 100 nL protein solution plus 100 nL reservoir solution. The commercial kits Crystal Screen and Index (Hampton) were used as initial conditions. Crystals were grown at 18 °C between 3 and 7 days, and screened for diffraction.

Crystals were supplemented with cryoprotection solution, flash cooled in liquid nitrogen and diffraction data were collected at 100 K, at beamline ID23-1 (wavelength of 0.97 Å) from the European Synchrotron Radiation Facility (Grenoble, France). A crystal grown in condition containing 22.5% PEG 4000, 14% isopropanol and 0.1 M sodium citrate, pH 6.0, was selected to collect diffraction data to 1.7 Å resolution. The complex of the enzyme with substrate was obtained by crystal soaking with five times molar excess of cellopentaose for 24 hours. Diffraction data for the complexed enzyme were collected at 1.75 Å resolution.

Data were integrated with iMosflm^34^ and scaled with Aimless^35^. The structure was solved by molecular replacement with Phaser^36^ using an endoglucanase from *Clostridium cellulovoran* (PDB code: 3NDY) as the search model. Coot^37^ was used for density fitting, and refinement was performed with PHENIX^38^.

### Atomistic simulations

We took the *Bl*Cel5B structure complexed with cellotetraose as the starting configuration for the MD simulations. The missing residues were taken from the apo *Bl*Cel5B structure after structural alignment using the LovoAlign server^39^. Hydrogen atoms were then added according to the protonation states determined at the optimum pH of 4.0 using the H + + server^40^. The following residues were considered protonated: H55, H77, D89, E96, E103, H114, E129, E159, E197, D198, E202, H205, E208, D211, H220, E245, E248, E260, H278, H292, D306, E312, E371, E375, E476, H416, E477, E489, D497, and E524. The remaining protonatable residues were considered in the standard protonation state. The *Bl*Cel5B-cellotetraose complex was then immersed in a rectangular simulation box of dimensions such that a solvent layer at least 16 Å thick surrounded the protein. The simulation box, built with Packmol^41^, also contained 0.10 M NaCl aqueous solution with excess counter ions to keep the system electrically neutral. The final system comprised approximately 85500 atoms.

The simulations were performed using NAMD^42^ with the CHARMM force field and the TIP3P water model^43^,^44^,^45^. Periodic boundary conditions were employed, using particle mesh Ewald^46^ to handle electrostatics and a 12-Å cutoff radius for truncating short-range potentials. Bonds involving hydrogen atoms were constrained at their equilibrium lengths and a time step of 2 fs was used to integrate the equations of motion. The simulations were carried out under constant pressure and temperature of 1 atm and 310 K, respectively, employing the Langevin barostat and thermostat^42^.

### Accelerated Molecular Dynamics

In accelerated molecular dynamics^20^,^47^, the trajectory is propagated on a modified potential aimed to enhance conformational sampling. Whenever the potential energy drops below a given threshold *E*, a boost Δ*V*(***r***) is applied, so that the escaping rates of local minima increase. When the potential energy gets over the threshold *E*, the system evolves on the original energy surface. This method has the advantage of conserving the general shape of the potential energy surface and of requiring no prior definition of reaction coordinates, so the system is allowed to explore freely its conformational space. Here, we restricted the energy boost only to the dihedral potential energy, as changes in torsion angles are the main source of conformational changes in proteins. The energy boost assumes the form of equation (1) that depends on the energy threshold *E* and on the parameter α – which modulates the shape of the potential energy surface where the boost is applied. We set the parameters *E* and α according to previous studies^48^, which recommend that *E* equals the average dihedral energy obtained from a conventional MD simulation plus 4 kcal/mol times the number of residues, and α equals 0.8 kcal/mol times the number of residues. The average dihedral energy was 2275.5 kcal/mol and the *Bl*Cel5B has 516 residues, so we set *E* = 2275.5 + 4 × 516 = 4339.5 kcal/mol and α = 0.8 × 516 = 418.8 kcal/mol.


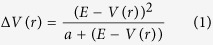


### Simulation procedures

*Bl*Cel5B-cellotetraose – Having built the system, we carried out the following steps for equilibration: (i) 1000 steps of energy minimization followed by 100 ps of MD simulation with all non-solvent heavy atoms fixed; (ii) same as (i), but with only the α carbons fixed; (iii) 5 ns of MD with all atoms free. After these preliminary steps, a trajectory lasting 400 ns was generated using conventional MD and then the aMD dihedral boost was applied for additional 1.0 μs. After 100 ns of conventional MD, the cellotetraose dissociated and the simulation began to represent the dynamics of unbound state of *Bl*Cel5B.

### *Bl*Cel5B-cellooctaose

To build the cellooctaose chain, we extended the original cellotetraose chain in the crystal structure with 4 additional glucose residues spanning regions around the *Bl*Cel5B positive subsites. Then, we submitted the system to the following procedure: (i) 1000 steps of energy minimization followed by 1 ns of MD keeping all the non-solvent heavy atoms fixed, except the 4 modeled glucose residues of the cellooctaose chain; (ii) same as step (i), but with only the α carbons fixed; (iii) 1 ns of MD with only the non-modeled glucose residues fixed. We then performed a 200-ns-long MD with three harmonic potentials involving cellooctaose chain: first, between C3 atom (CHARMM atom names) of the second glucose residue from the cellooctaose non-reducing end and the CD2 atom of the W47 tryptophan residue; second, between the OH3 atom of the forth glucose residue from the cellooctaose non-reducing end and HE2 atom of the H113 histidine residue; and third, between the HE2 atom of the catalytic residue E159 and O4 glycosidic oxygen between the fourth and fifth glucose unit of the cellooctaose chain. After these preliminary relaxation steps, the harmonic potentials were removed and the trajectory was propagated by 400 ns using MD. To get a model of the *Bl*Cel5B-cellooctaose complex in the closed conformation, we took the configuration after 80 ns of the restrained 200-ns MD simulation as the starting point for a 500-ns-long restrained aMD simulation, in which the CBM46 moved towards the CD in the presence of the harmonically-restrained cellooctaose chain. After this procedure, we released the restraints and propagated the closed *Bl*Cel5B-cellooctaose complex for additional 500 ns of conventional, restraint-free MD simulation.

### Coarse-grained MD simulations

The coarse-grained model was constructed from the minimized all-atom protein. We have used the domELNEDIN CG model for the protein. In this representation, an elastic network is used within each domain as a structural scaffold in order to maintain the overall shape of the protein, and a slightly modified version of MARTINI CG model describes the interactions involving beads not connected by harmonic springs^49^,^50^,^51^.

The delimitation of each domain was quite clear considering the short linkers connecting them and the recognition of their structural patterns in databases. We assumed CD, Ig-like module, and CBM46 as consisted of residues 18–331, 332–430, and 431–533, respectively. Therefore, there were elastic network bonds only within these domains (domELNEDIN CG model in Supplementary Fig. 6A).

The protonation state of each residue bead in the protein was the same adopted in the atomistic simulations. The system was then solvated by 10000 standard MARTINI CG water beads, including 10% of antifreeze particles. Also, 58 chloride and 48 sodium ions were added for charge neutrality. The size of final system was 109 Å × 109 Å × 109 Å.

Preliminary simulations were performed to test the elastic network (EN) parameters. We have tested six different ENs in 100 ns of simulations, using combinations of cut-off distance (*R*_c_) of 8 Å and 9 Å with spring force constant (*k*_*s*_) of 500, 800 and 1000 kJ mol^−1^ nm^−2^. The time evolution of root mean square deviation relative to the crystal structure as well as the mobility profile of the protein in these simulations were compared to the correspondent data from a 100 ns atomistic simulation. From this procedure, the parameters *R*_c_ = 9 Å and *k*_s_ = 500 kJ mol^−1^ nm^−2^ resulted in the best match between atomistic and coarse-grained simulations (Supplementary Fig. 6B).

The coarse-grained simulations were carried out using GROMACS^52^. Periodic boundary conditions were employed. Van der Waals interactions were shifted to zero in the range 0.9–1.2 nm, and the electrostatic interactions, in the range 0.0–1.2 nm. The simulations were performed in the isothermal-isobaric ensemble (NpT), employing the Berendsen thermostat and barostat for temperature and pressure control, respectively, with time constants τ_T_ = 0.5 ps and τ_p_ = 1.2 ps.

The CG simulations were carried out using the following protocol: the system was first minimized for 1000 steps using the steepest descent method. Then, it was submitted to a relaxation procedure comprising gradual increasing in time step or temperature. In the first stage of relaxation, the protein beads were restrained with a 1000 kJ mol^−1^ nm^−2^ force constant and a 50 ps simulation was carried out at 50 K, using the short time step of 1 fs. In the second stage, the time step was increased up to 5 ps lasting 1000 ps of simulation time. In the last stage of relaxation, all the system is released to move and it underwent a gradual increase in temperature, consisting on five segments of 100 ps at 50, 100, 150, 200 and 310 K. After achieving the desired temperature of 310 K, we performed three production simulations using 20-fs timestep. We have used a random number generator for assigning velocities to generate three independent simulations.

In general, smoothing of the energy surface in CG model makes the time scales faster. A speed up factor of 4 is typically employed to rescale the time scale of MARTINI CG systems^51^. Therefore, all CG simulations times described here and in the main text are effective times, i.e., 4× simulation time.

### Small Angle X-ray Scattering

SAXS data were collected at the SAXS2 beamline of the Brazilian Synchrotron Light Laboratory-LNLS (Campinas, Brazil) on a bi-dimensional position sensitive CCD detector (MarResearch, USA) using the radiation wavelength 1.54 Å. The sample-detector distance of 1000 mm allowed covering the momentum transfer range 0.01 Å^−1^ < q < 0.35 Å^−1^ (*q* = 4*π*sin *θ*/*λ*, where 2*θ* is the scattering angle).

The protein samples were prepared in McIlvaine’s buffer at 50 mM, pH 5 and 20 °C^53^. In each measurement, two successive frames of 300s were recorded for each sample at 1 and 2 mg/mL to monitor radiation damage. The patterns were integrated using the FIT2D program^54^. The comparative analysis for each scattering curve at 1 and 2 mg/mL of *Bl*Cel5B (data not shown), as well as the radius of gyration values (Rg), indicated that concentration and aggregation effects did not exist. The linearity of the Guinier plot indicated that the preparation was monodisperse.

The radius of gyration of the molecules (R_g_) was estimated by two methods, using the Guinier equation-*I*(*q*) = *I*(0).exp[(−*q*^2^.*R*_*g*_^2^)/3], *q.R*_*g*_ < 1.3- and also with the inverse Fourier transform in GNOM^55^. The same program was used to obtain the distance distribution function P(r) and the maximum diameter D_max_. Ten independent dummy atom models (DAMs) were restored by the *ab initio* proceeding implemented in DAMMIN package^56^. The best model, selected using normalized spatial discrepancy parameter computed by DAMAVER program, was superimposed on the crystallographic model with the SUPCOMB.

Then, based on the enzyme conformations reported by MD and protein crystallography, the computed X-ray scattering profile was fitted to a given experimental SAXS data by minimizing the *χ* function in the FOXS program^57^.

To assess the inter-domain information, the contribution of individual conformer and the flexibility of *Bl*Cel5B, we proceed in two approaches. First, the theoretical profiles and experimental data comparison was performed to infer the best-fit conformation of the ensemble-based analysis by the ensemble optimization method – EOM^21^, which assumes coexistence of a range of conformations in solution for which an average scattering intensity fits the experimental SAXS data; all models were generated with the three individual domains (Ig-like, CBM46, and CD) free to randomly move in order to cover the entire conformational space. The second approach was based on a fractional volume calculation from three conformation members extracted from the MD simulations, each with a distinct scatter curve. OLIGOMER^58^ provided solution of a system of linear equations between the experimental and generated conformations by MD.

The simulated scattering curves from the MD and crystallographic models were obtained using the CRYSOL^59^.

### Phylogenetic assignment

Sequences for all GH5 members, in which only the catalytic domain were considered, were downloaded from PFAM database^24^ and their classification into subfamilies were obtained within the CAZy database^23^. The sequences belonging to subfamily 4 were selected and those that had over 90% identity or represented partial coverage were rejected. Based on their multiple sequence alignment, the phylogenetic tree was constructed using the maximum likelihood method implemented in the MEGA program version 6.06^60^. One hundred Bootstrap replications were performed to examine the reliability of the phylogenetic tree.

## Additional Information

**How to cite this article**: Liberato, M. V. *et al.* Molecular characterization of a family 5 glycoside hydrolase suggests an induced-fit enzymatic mechanism. *Sci. Rep.* **6**, 23473; doi: 10.1038/srep23473 (2016).

## Supplementary Material

Supplementary Information

Supplementary Video S1

## Figures and Tables

**Table 1:** Activity of *Bl*Cel5B constructs against tested substrates.

Substrate (1%)	Relative Activity (%)				
	WT*	W479A	W481A	ΔCBM46	ΔIg-CBM46
β-glucan	100	79.1	63.6	nd	nd
CMC	25.5	12.2	2.4	nd	nd
Lichenan	52.4	41	28.6	nd	nd
Xyloglucan	45.2	41.2	30.8	nd	nd
Azo-Avicel	nd**	nd	nd	nd	nd
Arabinoxylan	nd	nd	nd	nd	nd
Galactomannan	nd	nd	nd	nd	nd
1,4-β-mannan	nd	nd	nd	nd	nd

^*^WT = wild type.

^**^nd = not detected.
