# Corrigendum: Molecular characterization of a family 5 glycoside hydrolase suggests an induced-fit enzymatic mechanism

**DOI:** 10.1038/srep36428

**Published:** 2016-12-14

**Authors:** Marcelo V. Liberato, Rodrigo L. Silveira, Érica T. Prates, Evandro A. de Araujo, Vanessa O. A. Pellegrini, Cesar M. Camilo, Marco A. Kadowaki, Mario de O. Neto, Alexander Popov, Munir S. Skaf, Igor Polikarpov

Scientific Reports 6: Article number: 23473; 10.1038/srep23473 published online: 04 01 2016; updated: 12 14 2016.

In this Article, the legend of Figure 5 is incorrect:

(**a**) BlCel5B in the crystallographic and closed configuration; (**b**) Bacillus halodurans Cel5B (BhCel5B) (PDB id: 4V2X) (**c**) Piromyces rhizinflata GH5 endoglucanase (PDB id: 3AYR); (**d**) Clostridium cellulolyticum GH5 endoglucanase (PDB id: 1EDG); (**e**) Clostridium cellulovorans GH5 endoglucanase (PDB id: 3NDY); (**f**) Bacteroides ovatus GH5 xyloglucanase (PDB id: 3ZMR); (**g**) Paenibacillus pabuli GH5 xyloglucanase (PDB id: 2JEP); (**h**) Prevotella bryantii GH5 endoglucanase (PDB id: 3VDH); (**i**) Ruminiclostridium thermocellum multifunctional GH5 cellulase, xylanase and mannase (PDB id: 4IM4); (**j**) Bacteroidetes bacterium AC2a endocellulase (PDB id: 4YHE).

should read:

The main difference between BlCel5B and BhCel5B is that the latter exhibits a deeper cleft due to the presence of residue W181 in the loop between F177 and R185. We conjecture that this difference in the binding site architecture relates to the importance that the CBM46 plays in the BlCel5B enzymatic mechanism.

In addition, the legend of Figure 6 is incorrect:

The main difference between BlCel5B and BhCel5B is that the latter exhibits a deeper cleft due to the presence of residue W181 in the loop between F177 and R185. We conjecture that this difference in the binding site architecture relates to the importance that the CBM46 plays in the BlCel5B enzymatic mechanism.

should read:

(**a**) BlCel5B in the crystallographic and closed configuration; (**b**) Bacillus halodurans Cel5B (BhCel5B) (PDB id: 4V2X) (**c**) Piromyces rhizinflata GH5 endoglucanase (PDB id: 3AYR); (**d**) Clostridium cellulolyticum GH5 endoglucanase (PDB id: 1EDG); (**e**) Clostridium cellulovorans GH5 endoglucanase (PDB id: 3NDY); (**f**) Bacteroides ovatus GH5 xyloglucanase (PDB id: 3ZMR); (**g**) Paenibacillus pabuli GH5 xyloglucanase (PDB id: 2JEP); (**h**) Prevotella bryantii GH5 endoglucanase (PDB id: 3VDH); (**i**) Ruminiclostridium thermocellum multifunctional GH5 cellulase, xylanase and mannase (PDB id: 4IM4); (**j**) Bacteroidetes bacterium AC2a endocellulase (PDB id: 4YHE).

