# Supplementary material for: Molecular characterization of a family 5 glycoside hydrolase suggests an induced-fit enzymatic mechanism
Source: Sci Rep. 2016 Apr 1;6:23473. doi: 10.1038/srep23473 (PMC4817029; doi:10.1038/srep23473)
Supplement: Supplementary Information [file srep23473-s1.pdf]

Supplementary Information

Molecular characterization of a family 5 glycoside hydrolase suggests an induced-fit enzymatic mechanism

Marcelo V. Liberato[a,#], Rodrigo L. Silveira[b,#], Érica T. Prates[b], Evandro A. de Araujo[a], Vanessa O. A. Pellegrini[a], Cesar M. Camilo[a], Marco A. Kadowaki[a], Mario de O. Neto[c], Alexander Popov[d], Munir S. Skaf[b,*] and Igor Polikarpov[a,*]

[a] São Carlos Institute of Physics, University of São Paulo, São Carlos 13566-590 São Paulo, Brazil

[b] Institute of Chemistry, University of Campinas, Campinas 13083-970, São Paulo, Brazil

[c] Institute of Bioscience, University of São Paulo State, Botucatu 18618-970, São Paulo, Brazil

[d] European Synchrotron Radiation Facility, Grenoble CS40220, France

[#] These authors contributed equally.

* Corresponding authors:

Munir S. Skaf, Institute of Chemistry, University of Campinas, Cx. P. 6154 Campinas, SP, 13084-862, Brazil

Phone: +55(19)3521-3093; E-mail: skaf@iqm.unicamp.br and

Igor Polikarpov, São Carlos Institute of Physics, University of Sao Paulo, Av. Trabalhador são-carlense, 400 Pq. Arnold Schimidt, São Carlos, SP, 13566-590, Brazil

Phone: +55(16) 3373-8088; E-mail: ipolikarpov@ifsc.usp.br

SUPPLEMENTARY FIGURES

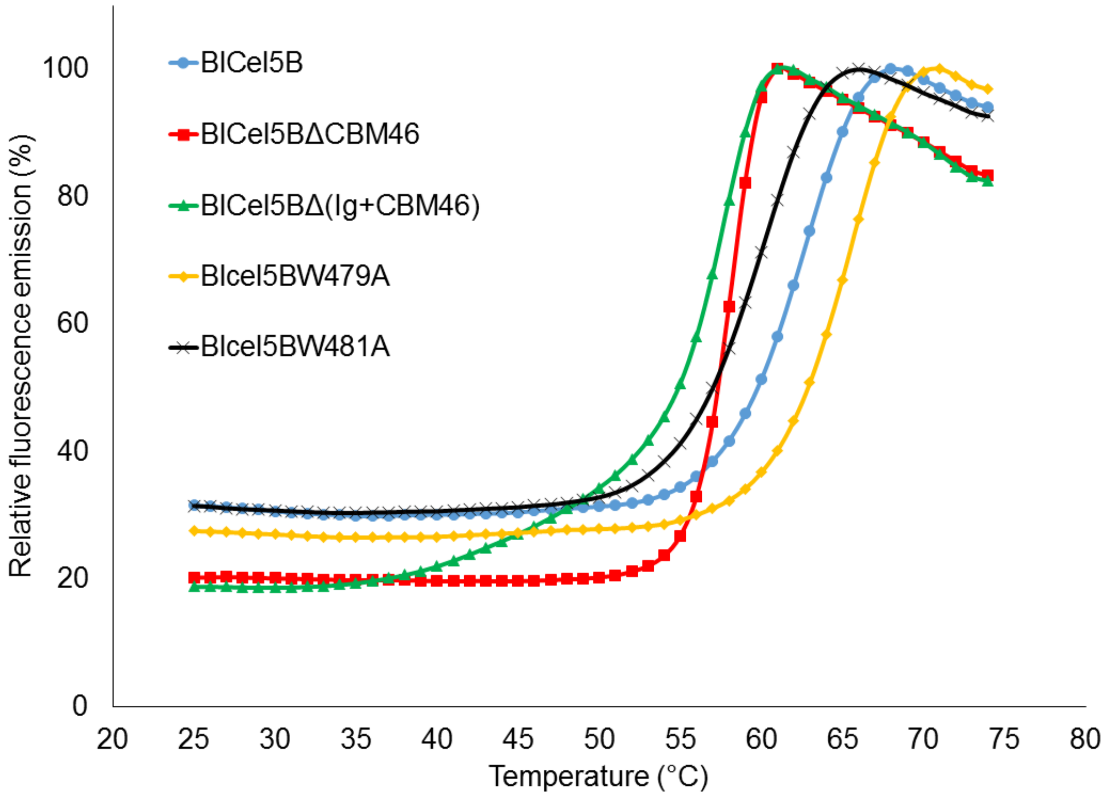

Supplementary Fig. 1 | Effect of deletions and point mutations on *Bl*Cel5B thermostability. Thermal Shift Assay was applied to evaluate possible enzyme destabilization caused by deletions and site-directed point mutations. All of the constructs have melting temperature higher than 55 °C, which is the optimum temperature for *Bl*Cel5B activity.

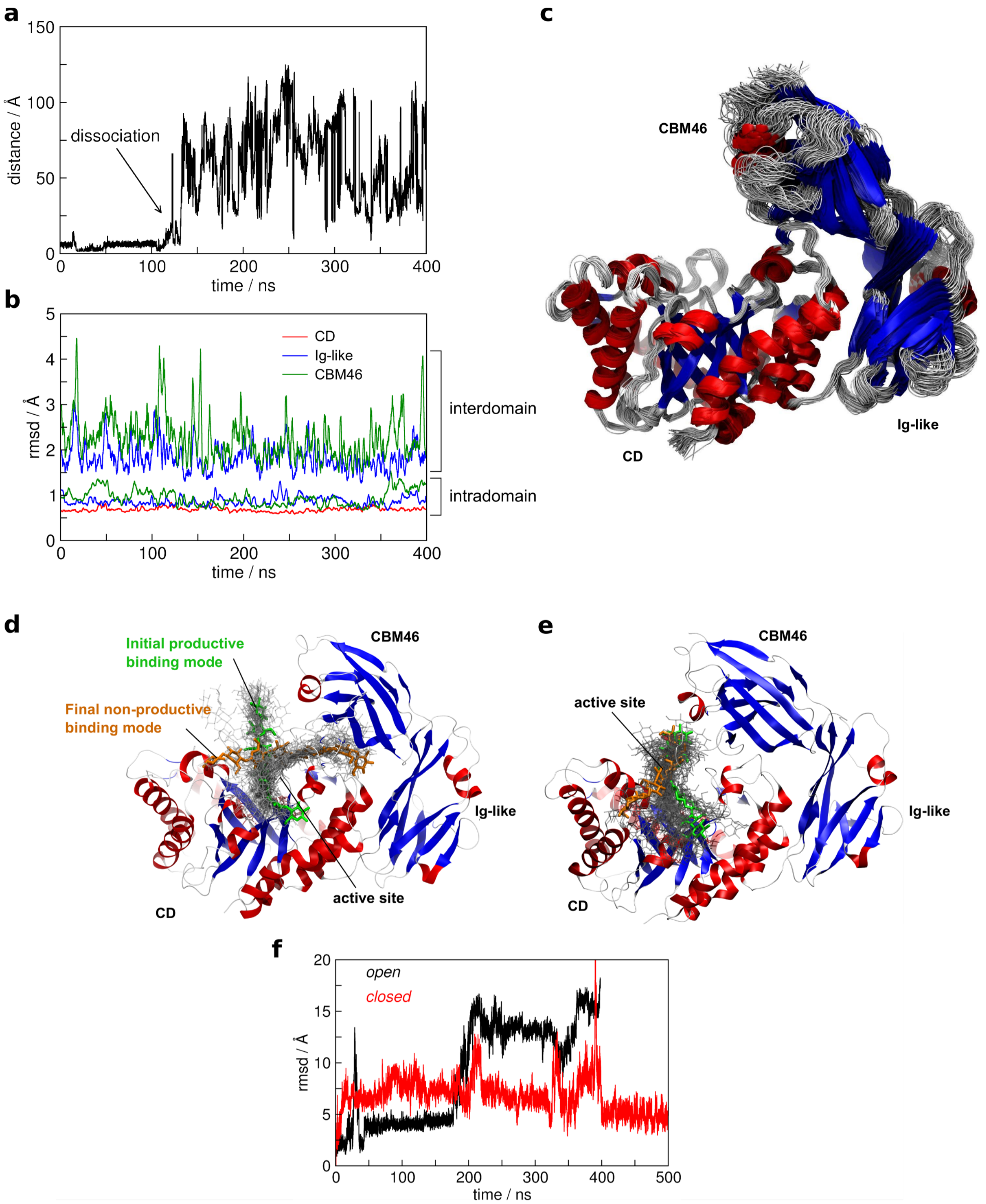

Supplementary Fig. 2 | Molecular dynamics of *Bl*Cel5B. (a) Rmsd of the cellotetraose chain during a conventional molecular dynamics simulations strarting from its configuration in the *Bl*Cel5B crystal structure. The rmsd – computed after alignment of the *Bl*Cel5B CD – fluctuates around low and constant values until ~100 ns, indicating that the cellotetraose remains bound to the enzyme during this time. After 100 ns, the rmsd increases, indicating that the substrate left the enzyme and started exploring the bulk. **(b)** α carbon rmsd relative to the average structure of the domains CD, Ig-like module and CBM46. The intradomain

rmsd's were computed after structural alignment of the same domain for which the rmsd was computed. As these intradomain rmsd's fluctuates around 1 Å, the *Bl*Cel5B domains do not exhibit conformational changes. The interdomain rmsd's were computed for each AM after structural alignment of the CD α carbons. The higher values of interdomain rmsd's compared to the intradomain rmsd indicates that the *Bl*Cel5B is more prone to exhibit large-scale interdomains than internal conformational changes. Running averages were performed on time windows of 2 ns for smoothing. **(c)** Dynamic picture of the *Bl*Cel5B, showing superposed configurations of the enzyme taken every 5 ns from a 400-ns MD simulation. Before the superposition, the CD was aligned so that the interdomain motions between the AMs became evident. As the substrate dissociated in 100 ns, this picture is representative of the substrate-free *Bl*Cel5B. **(d)** Dynamic picture of a cellooctaose chain (in gray sticks) in the open, crystallographic-like configuration of *Bl*Cel5B, showing that the substrate, initially in a productive binding mode (in green), assumes a non-productive binding mode (in orange) by the end of a 400-ns MD simulation, which is nearly perpendicular to the initial configuration and away from the active site. **(e)** Same as **(d)**, but after the *Bl*Cel5B transited to the closed configuration through AMD. In the closed configuration, the substrate is confined in the space between the flat binding surface of the CD andCBM46 lying over it. **(f)** Rmsd of the cellooctaose chain from its initial configuration in the simulations, showing that the closed *Bl*Cel5B configuration reduce the substrate freedom to move away from the initial, productive binding mode.

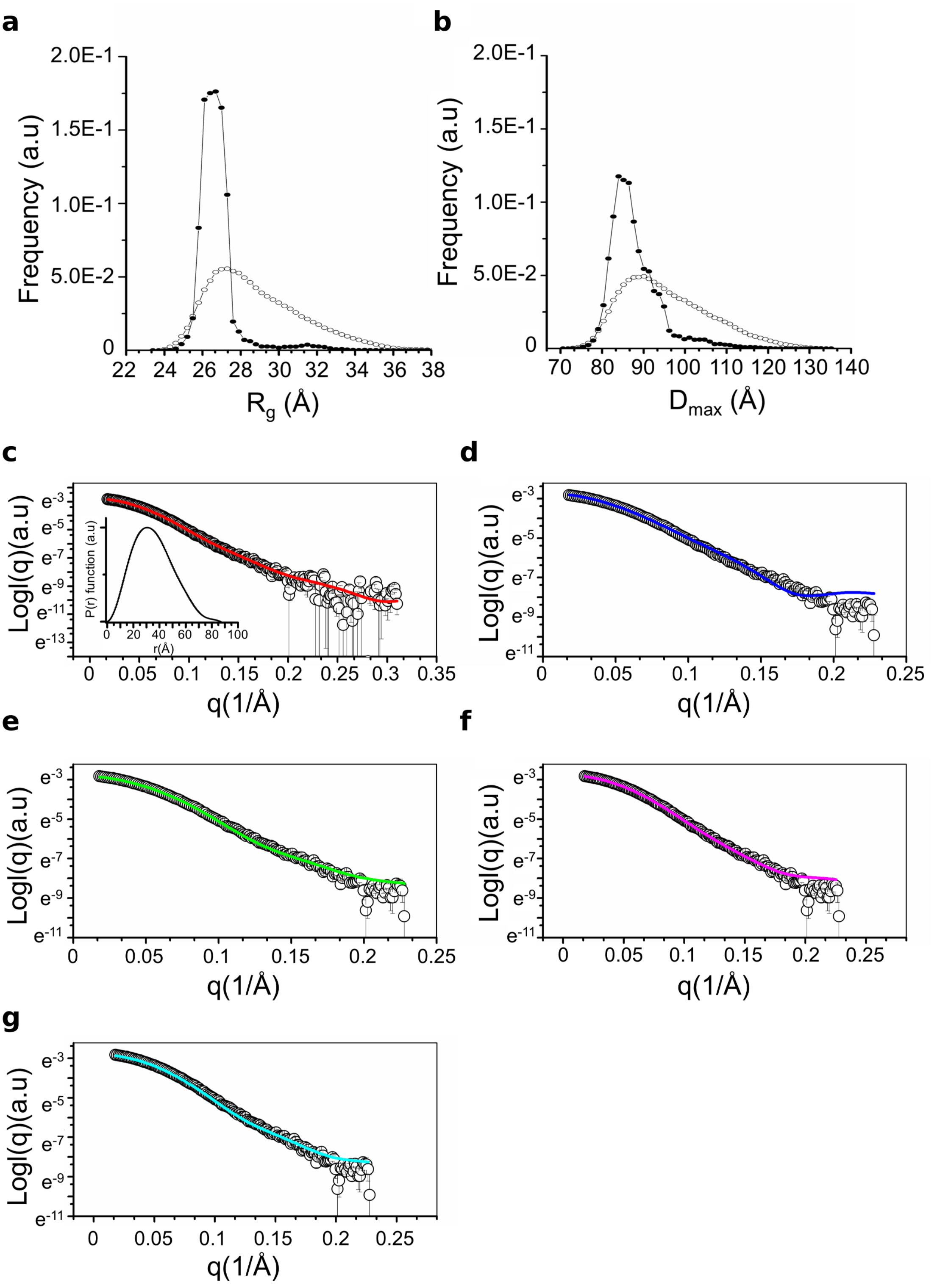

Supplementary Fig. 3 | Conformational distribution and *Bl*Cel5B SAXS data. Given the significant conformational flexibility of *Bl*Cel5B, we used EOM to analyze the distribution of different conformers of the protein. The R_g **(a)** and D_{max} **(b)** distributions reflect the overall size and shape properties of the molecules in solution. An initial ensemble (10,000 randomized models) of *Bl*Cel5B (open circles) with different conformations were generated and the scattering profiles of these ensemble were computed using EOM. The genetic

algorithm employed in EOM was used to select more representative structures (black circles) from the initial pool such that the average calculated scattering from the selected set of more representative conformations fits best the SAXS data. The R_g and D_{max} distributions reflect the relative populations of inter-domain conformational distributions (from extended to compact configuration) of *Bl*Cel5B in solution. **(c)** Experimental SAXS intensity is given as black open circles with errors bars and simulated SAXS scattering computed from DAM model is shown as red line. Inset, the distance distribution functions p(r) for BlCel5B. *Bl*Cel5B experimental SAXS data fitted with the theoretical scattering profile of the atomic structure from coarse-grained MD models: **(d)** open model scattering is given as a blue line; **(e)** closed model scattering is shown as a green line; **(f)** intermediate model scattering is presented as a pink line; **(g)** average model computed with OLIGOMER is shown as a cyan line.

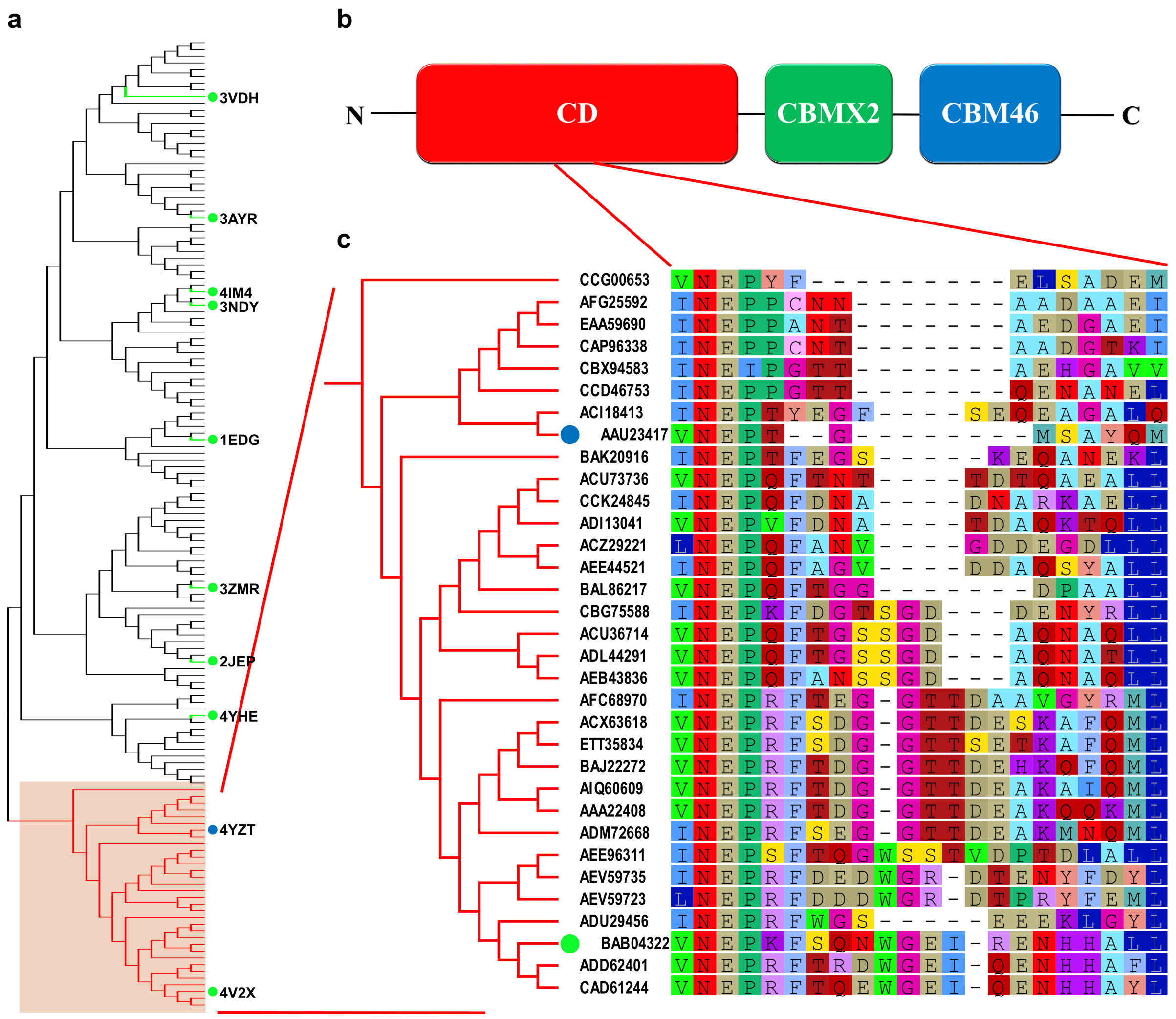

Supplementary Fig. 4 | Phylogeny of GH5 subfamily 4 (GH5_4). (a) The phylogenetic tree was constructed using all GH5 sequences assigned in CAZY to the subfamily 4, excluding the partial sequences and those that share identity greater than 90%. The members with the known crystallographic structure are highlighted with a green sphere followed by the PDB id code. The blue sphere represents *Bl*Cel5B**. (b)** Architecture representation of several GH5_4 enzymes, including *Bl*Cel5B. **(c)** Highlighted sub-tree composed by all GH5_4 members with the tri-modular architecture described in **(b)**. Each member is represented by its Genbank number. Alignment of the sequences evidencing the length of the loop between the residues P160 and M163 (with *Bl*Cel5B as a reference) is given at the right-hand side. First sub-group includes *Bl*Cel5B (blue sphere) and have a small loop, the second sub-group have an intermediary loop, and the third subgroup includes *Bh*Cel5B (green sphere) and have a long insertion loop.

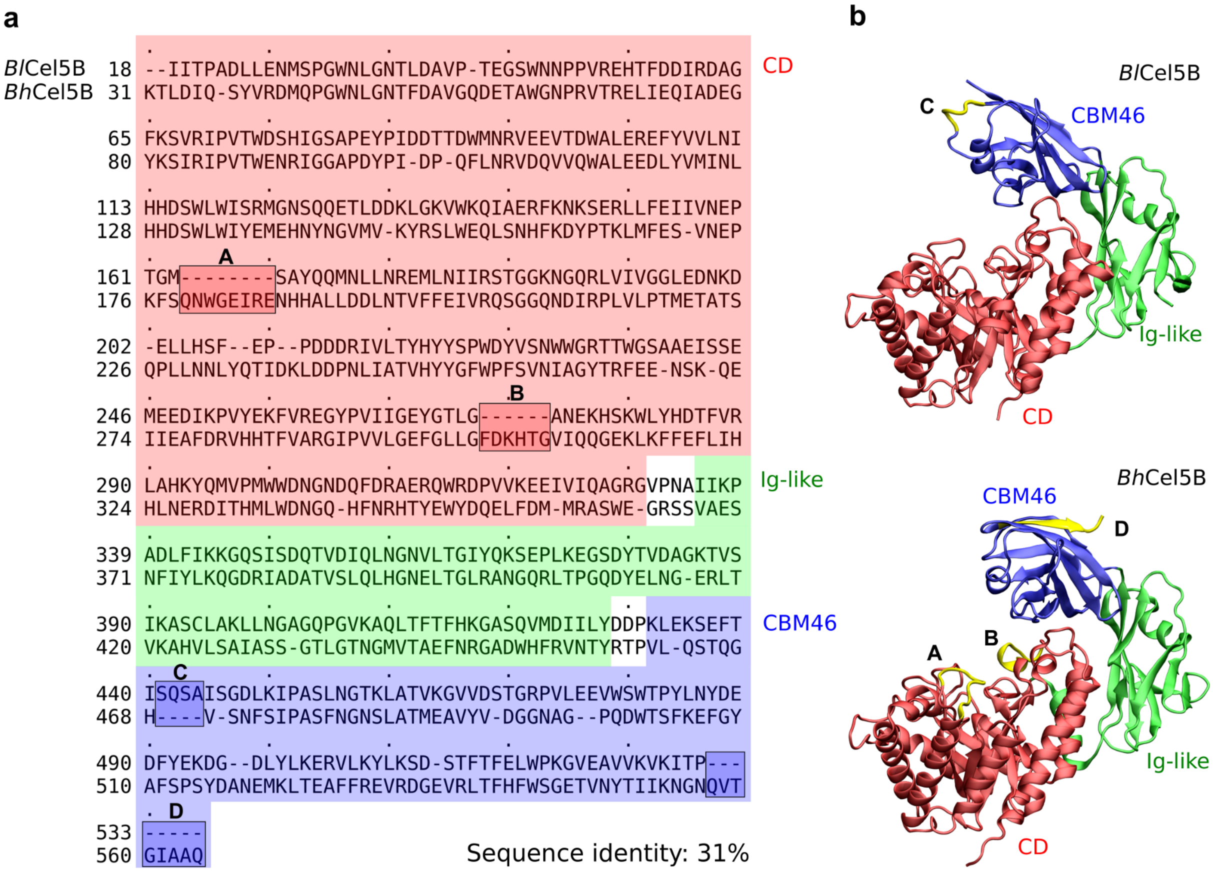

Supplementary Fig. 5 | Comparison between *Bl*Cel5B and *Bh*Cel5B. (a) Sequence alignment, with insertions/deletions highlighted and labeled as A, B, C and D. **(b)** Structure of *Bl*Cel5B and *Bh*Cel5B, with the insertions highlighted in yellow. The insertion A is a loop that shapes the *Bh*Cel5B binding cleft. The insertion B is located below the CBM46 in *Bh*Cel5B, and might be important for the relative mobility of CD and CBM46 in *Bh*Cel5B structure. The insertion C is located in the *Bl*Cel5B CBM46 and does not face the binding cleft. The insertion D is a C-terminal prolongation of the *Bh*Cel5B CBM46 and does not face the binding cleft. All other regions of *Bl*Cel5B and *Bh*Cel5B from the structural viewpoint are nearly identical.

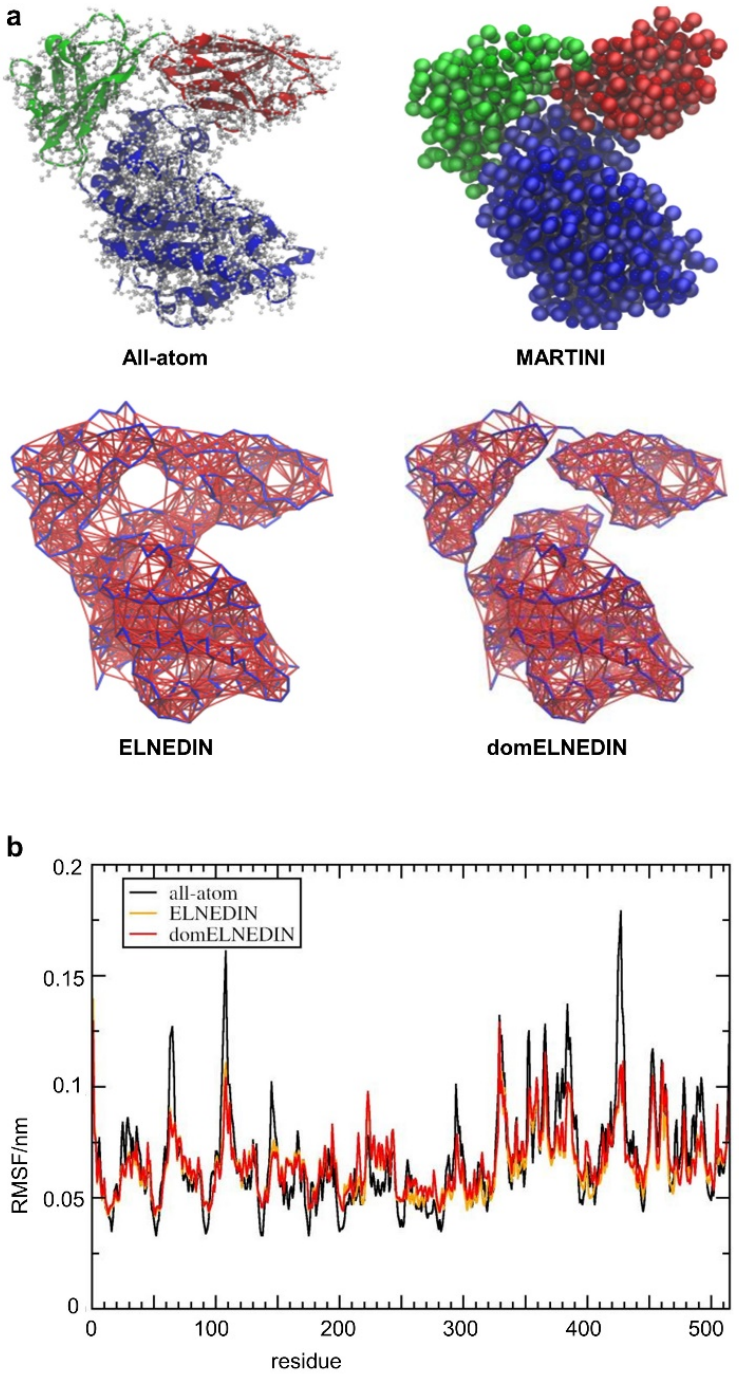

Supplementary Fig. 6 | Tested coarse-grained protein models. (a) Different representations of *Bl*Cel5B: atomistic, MARTINI coarse-grained, elastic networks (ELNEDIN) and intradomain elastic networks (domELNEDIN). In this study, we have performed simulations using all-atom (atomistic) and domELNEDIN coarse-grained models. **(b)** Comparison between the mobility profiles of *Bl*Cel5B in 100-ns all-atom MD simulation and the MARTINI-elastic networks simulations (ELNEDIN and domELNEDIN).

SUPPLEMENTARY TABLES

Supplementary Table 1 | Statistics for crystallographic data collection and refinement.

	*Bl*Cel5B+cellotetraose	*apo Bl*Cel5B
**Data collection**		
Space group	$P4_32_12$	$P4_32_12$
Cell dimensions a, b, c (Å)	91.3, 91.3, 124.7	90.7, 90.7, 120.6
Resolution (Å)	73.66-1.66 (1.69-1.66) *	64.13-1.7 (1.73-1.7)
R_{merge}	0.146 (0.802)	0.078 (0.552)
$I / \sigma I$	6.9 (2.2)	7.1 (1.7)
Completeness (%)	99.8 (99.9)	94.1 (95.9)
Redundancy	5.0 (5.2)	2.5 (2.5)
**Refinement**		
Resolution (Å)	57.33-1.67	64.13-1.7
# of reflections	62078	52066
R_{work} / $R_{free,}$	0.175 / 0.212	0.166 / 0.198
# of atoms		
Protein	4108	4040
Ligand/ion	53	0
Water	738	593
B-factors		
Protein	19.3	21.8
Ligand/ion	22.2	
Water	32.8	35.2
R.m.s. deviations		
Bond lengths (Å)	0.007	0.007
Bond angles (°)	1.09	1.06
Ramachandran		
Favored	97.0	97.0
Allowed	2.81	2.8
Outliers	0.19	0.2

* Highest resolution shell is shown in parentheses. Both structures are based on single crystals.

Supplementary Table 2 | SAXS data and analysis.

Data-collection parameters				
Wavelength	1.54 Å			
q range	0.015 to 0.338 $Å^{-1}$			
Exposure time	300 s			
Concentration	2 mg/mL			
Temperature	20 °C			
**Structural parameters**		**SAXS**	**Crystal/Intermediate**	
R_g (Å) from Guinier plot		27.40 ± 0.06	None	
R_g (Å) from P(r)		27.10 ± 0.03	24.99	
D_{max} (Å)		87.00	78.00	
Fitting SAXS profile by minimizing χ function using experimental data				
$\chi_{Exp/DAM}$	1.40			
Fitting SAXS profile by minimizing χ function between experimental data and MD models				
	Intermediate	Closed	Open	Average
χ χ	2.62	3.27	4.45	1.89

Supplementary Table 3 | Sequences of the primers used in *Bl*Cel5B constructs cloning.

Primer name	Sequence (5´- 3´)
*Bl*cel5B_Fw	CAGGGCGCCATGGTTCCGAAAGCTTCGGG
*Bl*cel5B_Rv	GACCCGACGCGGTTAAGGCGTGATTTTCACCTTCACG
*Bl*cel5BΔ1006-1602_Rv	GACCCGACGCGGTTAGATCGCATTGGGAACGC
*Bl*cel5BΔ1297-1602_Rv	GACCCGACGCGGTTACTTCGGGTCATCATAAAGAATGATATC
*Bl*cel5BW479A_Fw	TTGAAGAAGTAGCGTCATGGACG
*Bl*cel5BW479A_Rv	GAACCGGCCTTCCTGTAGAATC
*Bl*cel5BW481A_Fw	AGTATGGTCAGCGACGCCCTAC
*Bl*cel5BW481A_Rv	TCTTCAAGAACCGGCCTTCC

SUPPLEMENTARY VIDEO S1 | Video obtained from the molecular trajectories generated by MD simulations of the substrate-free BlCel5B showing open-close transition of the CBM46 over the catalytic domain (CD).
